# Predictors of successful outcome after adrenalectomy for unilateral primary aldosteronism

**DOI:** 10.3389/fendo.2023.1205988

**Published:** 2023-08-11

**Authors:** Ahmed Saadi, Mohamed Ali Bedoui, Selim Zaghbib, Hamza Boussaffa, Seif Mokaddem, Ibtissem Ben Nacef, Haroun Ayed, Amine Derouiche, Karima Khiari, Marouene Chakroun, Riadh Ben Slama

**Affiliations:** ^1^ Department of Urology, University of Medicine of Tunis, Charles Nicolle Hospital, Tunis, Tunisia; ^2^ Department of Endocrinology, University of Medicine of Tunis, Charles Nicolle Hospital, Tunis, Tunisia

**Keywords:** primary aldosteronism, adrenalectomy, hypertension, resistant hypertension, prediction systems

## Abstract

**Introduction:**

Unilateral primary aldosteronism (UPA) is the most frequent surgically curable form of endocrine hypertension. Adrenalectomy is the cornerstone of treatment for UPA, but outcomes after surgery are variable. The cause of resistant hypertension after surgery is still a matter of debate. Our aim was to investigate cure rates after surgery and to evaluate preoperative factors that might influence the surgical outcome.

**Methods:**

Between 2000 and 2021, the charts of 71 Tunisian patients who underwent laparoscopic adrenalectomy for UPA were retrospectively reviewed. Preoperative medical records were collected and follow-up data (1-158 months) were registered. Antihypertensive medication doses were calculated using defined daily doses (DDD) and postoperative outcomes were assessed using the Primary Aldosteronism Surgical Outcome (PASO) criterion.

**Results:**

Of 91 enrolled patients, 71 (59% women, mean age 46 years, median length of follow-up 21 months) were suitable for evaluation. Thirty-four patients (48%) had complete clinical success according to the PASO criteria. The most relevant factors associated with complete clinical success on univariate analysis were: absence of diabetes (p= 0.007), low body mass index (BMI) (p= 0.001), lower preoperative DDD (p= 0.01), preoperatively controlled blood pressure (p= 0.024), higher plasma aldosterone to renin ratio (ARR) (p= 0.001), adenoma subtyping (p <0.001) and aldosteronoma resolution score (ARS) (p= 0.002). Multivariate regression analysis showed that the major predictors of complete clinical success were absence of diabetes (OR: 5.205), a BMI < 30 (OR: 4.930), a plasma ARR > 332 (OR: 4.554) and an ARS ≥ 3 (OR: 2.056).

**Conclusion:**

Complete and partial clinical response rates were achieved in respectively 48 and 43% of cases. The main predictors of complete resolution of hypertension were absence of diabetes, low BMI, high plasma ARR and high ARS. Taking these factors into account may help identify patients at risk of persistent postoperative hypertension who may require long-term surveillance and medication.

## Introduction

Primary aldosteronism (PA) is caused by excessive, aberrant production of aldosterone by the cortex of one or possibly both adrenal glands (1). The current prevalence of this disease is estimated at around 5-13% in the high blood pressure population (2, 3). It is the most frequent etiology of secondary hypertension generally accompanied by hypokalemia (3, 4). Patients with PA have a two- to fivefold higher risk of cardiovascular disorders compared to patients presenting with essential hypertension (5). Over time, PA leads to fibrosis and remodeling of major organs, resulting in an elevated cardiovascular, renal and cerebrovascular morbidity and mortality (6–8). PA is caused by bilateral idiopathic adrenal hyperplasia which requires medical treatment with mineralocorticoid receptor antagonists (MRA) or by an adrenocortical adenoma, a condition that is potentially curable by surgery (9). There are different definitions of recovery from hypertension after PA surgery in the literature, and documented cure rates in different series vary from 16% to 72% (10, 11). Lately, there has been a proposal to establish standardized criteria for reporting outcomes after surgery for PA. The PASO (Primary aldosteronism surgical outcome) study (12) has established harmonized clinical and biochemical outcome parameters. In the case of unilateral dominant PA, the question is to identify a patient profile that would be qualified as a “good responder” to surgery through the identification of preoperative prognostic factors that are predictive of complete resolution of hypertension. The aim of this study was to investigate cure rates after surgery and to evaluate preoperative factors that might influence the surgical outcome.

## Materials and methods

Between January 2000 and December 2021, 71 consecutive patients treated by laparoscopic adrenalectomy for UPA at Charles Nicolle Hospital of Tunis were enrolled in this retrospective study. In our study patients were screened for PA if they presented one of the following conditions (8): hypertension (>140/90mmHg) resistant to three conventional antihypertensive drugs (including a diuretic) or controlled on four or more antihypertensive drugs, severe hypertension (systolic blood pressure ≥ 180mmHg and/or diastolic blood pressure ≥110mmHg), hypertension and permanent or intermittent hypokalemia, hypertension or hypokalemia with adrenal incidentaloma, hypertension and repercussions on target organs or cardiovascular morbidity disproportionate to the level of blood pressure and the duration of the progression of hypertension.

Medical records were reviewed and preoperative data including baseline characteristics, preoperative antihypertensive treatment, findings of imaging studies, and biochemical parameters were collected. Postoperative records consisted of surgical complications, hypertension outcome and histopathological findings.

The preoperative diagnosis of PA was determined by the presence of persistent hypertension with or without hypokalemia (<3.5mmol/L), with increased serum aldosterone concentration (≥200pg/mL), suppressed plasma renin concentration (≤5pg/mL; 5 pg/mL = 8.35 mIU/L) and aldosterone-to-renin ratio (ARR) (≥23). Samples were collected in the morning, more than 2 hours after rising, in a seated position for 5-15 minutes, on a normo-sodium and normo-kalemia diet, and after withdrawal of any medications interfering with the renin angiotensin system for at least 4 weeks before ARR testing. In the setting of spontaneous hypokalemia with plasma renin below detection levels and plasma aldosterone concentration ≥200pg/mL and ARR ≥23 we dit not use confirmatory testing which was the case of 80% of the patients, for the remaining 20% a captopril challenge test was carried out for case confirmation. All included patients had Computed tomography (CT) before adrenalectomy. If the opposite adrenal gland was abnormal, patients were selected to undergo adrenal venous sampling (AVS) or iodocholesterol scintigraphy to distinguish a lateralized aldosterone hypersecretion. Histopathology results after surgery were analyzed and histological subtype was collected. The outcome of unilateral adrenalectomy was evaluated in all subjects at the time of hospital release and postoperatively at the six-month follow-up.

The PASO criteria for clinical outcomes were used to assess the results of adrenalectomy (12): (1) Complete clinical success included patients whose blood pressure was < 140/90 mmHg without treatment; (2) Partial clinical success was defined by a blood pressure < 140/90 mmHg on antihypertensive treatment with a reduction in the number and/or dose of antihypertensive treatments after adrenalectomy (3) Absent clinical success included patients whose blood pressure remained high after surgery, using either the same number of antihypertensive treatments or a greater number.

For determining factors influencing surgical outcome, patients with complete clinical success were compared to patients with partial and absent success combined. Two preoperative scores that predict complete clinical cure in UPA post adrenalectomy were calculated according to their original description: The Aldosteronoma Resolution Score (ARS) (13) and the Primary Aldosteronism Surgical Outcome (PASO) score (14) (**Table 1**). The PASO score and defined daily dose (DDD) were calculated using the online PASO Calculator (https://github.com/ABurrello/PASO-Predictor/blob/master/00%20-%20PASO%20Predictor.xlsm;accessed on 01/04/2020).

**Table 1:** Preoperative unilateral primary aldosteronism scoring systems for predicting clinical cure post adrenalectomy.

System	ARS (12)		PASO (13)	
Maximum score	5		25	
Score predicting cure (or lack of)	≥4 (≤1)		≥20 (≤10)	
Components	Criteria	Score	Criteria	Score
Sex	F	1	F	3
	M	0	M	0
BMI (kg/m2)	≤25	1	<24	1.5
	>25	0	24-29.9	0.5
			≥30	0
Hypertension duration	≤ 6 years	1	≤ 120 months	7.5
	> 6 years	0	120-239 months	3.5
			≥ 239 months	0
Antihypertensives	Classes		DDD	
	≤ 2	2	< 3	6
	≥ 3	0	3-9	3
			≥ 9	0
Target organ damage	Not included		No	3
			Yes	0
Nodule size (mm)	Not included		≥ 20	4
			13 – 19	2
			< 13	0

All statistical tests were conducted using IBM SPSS version 25. The primary endpoint was postoperative hypertension. Univariate analyses and multivariate stepwise logistic regression were performed to evaluate the prediction effect of the combined clinicopathological variables. For these analysis, hypertension outcome was the dependant variable and the clinical, biochemical, radiological and pathological variables were independent potential predictor variables. P < 0.05 was considered statistically significant.

## Results

A total of 71 patients who had unilateral laparoscopic adrenalectomy for PA with unilateral adrenal lesion on CT scan were included. The cohort involved 42 women (59%), and the mean age at surgery was 46 years. Further baseline characteristics are displayed in **Table 2**. Prior to surgery, most patients had poor hypertension control (47/71, 66%), and average duration of hypertension was 82.9 ± 75.9 months (1-288). On cross-sectional imaging, the left adrenal was most commonly affected (68%). Two patients required further investigations: one of them required AVS and the other one iodocholesterol scintigraphy. Retroperitoneoscopic adrenalectomy was performed in 47 patients (66%) and transperitoneal laparoscopic approach was performed in 24 patients (34%). Only 2 patients required conversion to open adrenalectomy because of bleeding that required transfusion with a mean of 2.2 units of blood transfused. Mean operating time was 167 ± 47 minutes (75-330), and average postoperative stay was 2.6 ± 1.8 days (1-10). The overall postoperative complication rate (Clavien-Dindo ≥3a) was 4%. No mortalities occurred. Preoperatively, all our patients were on antihypertensive medications with a mean DDD of 2.7 (1-5). Mean systolic blood pressure (SBP) was 147 ± 21 mmHg and mean diastolic blood pressure (DBP) was 86 ± 13 mmHg. At the time of diagnosis, the average kalemia of our patients was 3.0 ± 0.7 mmol/L (1.6-5.1).

**Table 2:** Summary of study group characteristics.

Variable	Cohort (n=71)
Sex (male/female)	29/42
Age (y)	46 ± 11
Body mass index (kg/m2)	27.6 ± 4.4
Diabetes	19 (27%)
Duration of hypertension (months)	82.9 ± 75.9
Nodule size (mm)	22 ± 9
Side of the adrenalectomy (right/center)	23/48
Preoperative SBP (mmHg)	147 ± 21
Preoperative DBP (mmHg)	86 ± 13
Preoperative DDD of antihypertensive medications	2.7 ± 1.1
Preoperative serum potassium (mmol/L)	2.9 ± 0.7
Serum aldosterone (pg/mL)	534 ± 420
Plasma renin concentration (pg/mL)	4.2 ± 7.2
Median Plasma aldosterone-to-renin ratio (IQR)	155 (500)
ARS	2.5 ± 1.5 (0 – 5)
PASO score	17.3 ± 4.5 (3.5 – 25)
Surgical technique Retroperitoneal approach Transperitoneal approach	47 (66%) 24 (34%)
Operative time (min)	167 ± 47
Post-op complications, Clavien-Dindo ≥ 3a	3 (4%)
Postoperative length of hospital stay (d)	2.6 ± 1.8
Postoperative DDD of antihypertensive medications	1.8 ± 0.8
Postoperative SBP (mmHg)	129 ± 13
Postoperative DBP (mmHg)	78 ± 9
Final pathology Solitary adenoma Nodular hyperplasia	58 (82%) 13 (18%)

Histology revealed that 58 patients (82%) had solitary adenoma and 13 patients (18%) had adrenal hyperplasia. Mean adenoma size was 20.2 ± 9.9 mm (7-50).

**Figure 1:**
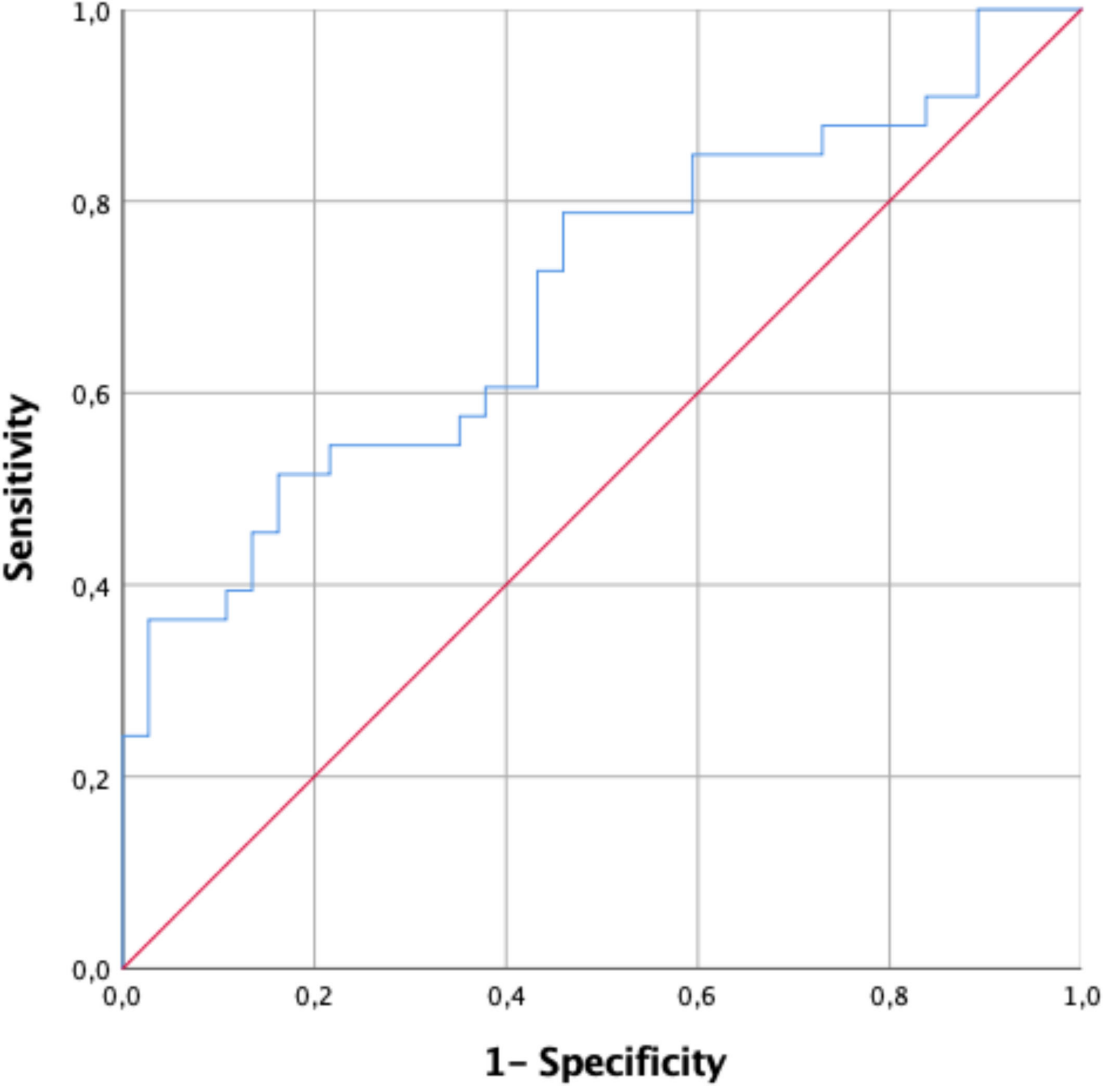
ROC Curve of the ARS applied to our patient cohort.

Median follow-up time was 6 months (range 1-158), at which time, according to the PASO criteria (11), 34 patients (48%) had complete clinical success, 30 patients (43%) had partial clinical success, and 7 patients (9%) had absent clinical success. The mean SBP and DBP of patients with complete clinical success were 118 ± 8 and 71 ± 7 mmHg. The same subgroup had a mean blood pressure of 144 ± 23/86 ± 13 mmHg before adrenalectomy. In patients with partial success, the mean DDD of antihypertensive medication was reduced from 3.3 to 1.6 (p = 0.001). The use of antihypertensive medication increased from a mean DDD of 2.5 to 3.7 (p = 0.07) after surgery in patients without absent clinical success. Based on preoperative clinical characteristics, the ARS was calculated. For our total cohort, the mean score was 2.5 (0-5). A receiver operating characteristic (ROC) curve was plotted and the area under the curve (AUC) was calculated to assess predictive accuracy of the ARS in our patients. The AUC for ARS was 0.707 (95% CI 0.586-0.828) (**Figure 1**). An ARS ≥ 3 represented the cut-off at which the probability of complete resolution of postoperative hypertension was the highest, with a positive predictive value of 65.7% and a negative predictive value of 69.4%.

**Table 3** summarizes the predictive factors of complete clinical success in univariate analysis. Patients with complete clinical success after adrenalectomy had a mean age of 44 years, compared to 47 years for those 37 patients with persistent hypertension (partial and absent clinical success) (p= 0.273). Patients with persistent post-operative hypertension were more likely to have type 2 diabetes (p = 0.007) and more likely to have a higher BMI (p = 0.001). In addition, patients with persistent hypertension were receiving more antihypertensive medications (p=0.01) and exhibited poor blood pressure control before surgery (p=0.024). Patients with continued postoperative hypertension had a significantly lower rate of ARR (p = 0.001) and a significantly lower ARS score (p = 0.002). They were also more often found to have hyperplasia than adenoma (p=0.043). Additional factors, such as female sex, a history of hypertension in the family, duration of hypertension, side and size of the lesion, serum potassium and the PASO score were not correlated with complete clinical success.

**Table 3:** Univariate analysis of clinical outcome according to candidate predictor variables.

	Complete clinical success, (n =34)	Partial and absent clinical success (n = 37)	P-value
Age (y)	44.2 ± 10,2	47.1 ± 11.8	0.273
Sex (female/male)	64.7%	54.1%	0.369
Diabetes (%yes)	11.8%	40.5%	0.007
Body mass index (kg/m2)	25.8 ± 3.1	29.5 ± 5.2	0.001
Family history of hypertension (%yes)	5.9%	16.2%	0.174
Duration of hypertension (mo)	77.9 ± 76.5	91.8 ± 74.4	0.443
Preoperative DDD of antihypertensive medications	2.4 ± 1.1	3.1 ± 1.0	0.01
Preoperative blood pressure control (% controlled)	47.1%	21.6%	0.024
Target organ damage (%)	26.5%	27%	0.959
Preoperative serum potassium (mmol/L)	2.9 ± 0.6	3.1 ± 0.8	0.289
Severe hypokalemia (%)	57.1%	51.4%	0.756
Serum aldosterone (pg/ml)	613 ± 445	517 ± 550	0.13
Plasme aldosterone-to-renin ratio	655 ± 706	214 ± 242	0.001
Nodule size (mm)	19.8 ± 10.3	20.4 ± 9.6	0.807
Histology (%solitary adenoma)	91%	73%	< 0.001
ARS score	3.1 ± 1.3	2.0 ± 1.5	0.002
PASO score	18.1 ± 4.2	16.5 ± 4.6	0.13

On multivariate analysis the main predictors of complete clinical success were absence of diabetes (OR:5.205, 95%CI 1.411-19.206), a BMI < 30 (OR:4.930, 95%CI 1.564-15.545), a plasma ARR > 332 (OR:4.554, 95%CI 1.563-13.263) and an ARS ≥ 3 (OR:2.056, 95%CI 1.210-3.491) (**Table 4**).

**Table 4:** Results of multivariate logistic regression analysis of the major predictors.

Variables	Odds ratio (95% CI)	P
Absence of diabetes	5.205 (1.411 - 19.206)	0.013
BMI < 30	4.930 (1.564 - 15.545)	0.006
Plasma ARR > 332	4.554 (1.563 - 13.263)	0.005
ARS ≥ 3	2.056 (1.210 - 3.491)	0.008

## Discussion

Several series have demonstrated the benefits of adrenalectomy in UPA, even in the presence of hyperplasia, by reducing blood aldosterone levels (12). The state of hyperaldosteronism induced by chronic autonomous glomerular secretion will be responsible for its inherent cardiovascular toxicity, independently of the severity of hypertension, leading to multiple complications such as myocardial infarction, atrial fibrillation, left ventricular hypertrophy, arterial stiffness, metabolic syndrome and renovascular alterations (6–8). Furthermore, a cost-effectiveness analysis performed for 50-year-old patients with PA revealed that adrenalectomy was a cost saving option thanks to lifelong drugs withdrawal, even in case of inclusion of patients with persistent hypertension after surgery (15).

This study shows that laparoscopic adrenalectomy is a reliable and efficient treatment option for lowering blood pressure in patients with UPA. In our study, complete clinical success was reported in 48% of the cases and partial clinical success in 43%. Whereas, absent clinical success rate was noted only in 9% of the patients.

Relevant and reproducible definitions of the clinical and biochemical outcome of surgical treatment have been made possible by the development of the PASO criteria (16). Unfortunately, the assessment of biochemical outcomes was hampered by missing data and may be partly explained by the lack of consistent reporting criteria during the study period (2000-2021), with the proposed PASO criteria being published in 2017 (12).

Complete resolution of hypertension was observed in 48% after surgery. This figure is similar to that found in previous studies (10, 11), but seems to be higher than the 37% rate found in the PASO study (12). If we compare our patients with those in the PASO group, we can see that the average amount of antihypertensive medication is slightly higher than in their group (mean DDD of 2.4 compared to 2.0). Except of a longer duration of known hypertension before surgery in our cohort compared to the PASO cohort (77 versus 60 months), other baseline characteristics, such as age and BMI, were comparable between studies. Partial clinical success was achieved in 43% of the cases which is slightly lower but almost equivalent with the PASO investigators result of 47%. Apart from a larger adrenal nodule diameter in our patients (25 versus 12 mm), there were no other baseline characteristics that showed significant differences between data that could explain this disparity. The absence of clinical response, estimated at 9% in our study, is much lower than the 16% found in the PASO study. In our study despite the fact that patients with continued hypertension (partial and absent clinical success groups) were slightly older and presented with a longer known duration of hypertension before surgery than the patients who responded positively to adrenalectomy, young age and shorter duration of hypertension were not associated with complete clinical success after surgery.

Indeed, many studies have suggested that patients are more likely to become normotensive after adrenalectomy if they are aged 50 years or younger and have had hypertension for less than 5 years at the time of surgery (12, 14, 17, 18). It has been suggested that long-lasting hypertension (despite surgical correction of hormonal abnormalities) causes irreversible vascular damages, which is the main reason for explaining why prolonged hypertension is a risk factor for surgical outcome (19). Higher BMI was associated with an increased likelihood of continued hypertension after surgery. Although this result is consistent with that found in similar studies (11–14, 18), the reason for this finding remains unclear but could be related to higher aldosterone levels in patients with obesity (20). Moreover, absence of diabetes was associated with complete resolution of hypertension. The rationale for this association may be that patients with diabetes have increased and permanent vascular damage over and above that caused by hypertension (21). In our study controlled blood pressure before surgery, an indication of lower disease severity, was associated with complete clinical success after surgery. Vascular remodeling may help explain why. A study has shown that in case of severe hypertension, the compensatory mechanism for counteracting the rise in blood pressure is less effective, leading to vascular damage (22).

An unanticipated and very interesting result which needs further investigation was that higher plasma aldosterone to renin ratio was associated with complete clinical success. An ARR > 332 constitutes the threshold value from which the prediction of cure of hypertension is the strongest. In a recent meta-analysis, the identification of this factor as a predictive parameter for cured hypertension after adrenalectomy could not be established (18). Nevertheless, this result was more balanced in a review of the literature involving 1486 patients where only 2 studies identified a high ARR, in univariate analysis, as a predictive factor of complete clinical success after surgery (11). Yang et al. (23) found a strong association between ARR and cure of hypertension, which led to this parameter being taken into account in a preoperative score predicting cure of hypertension after surgery. The hypothesis put forward by these authors, which we support, is that patients with a very high ARR at the time of diagnosis were at greater risk of presenting a more aggressive form of the disease with severe hypertension and pronounced hypokalemia, which would have made it possible to establish the diagnosis and treat these patients earlier.

In our study ARS ≥ 3 was found to be a predictive factor of cured hypertension after adrenalectomy. Without exception, ROC AUC was lower in our cohort than in its original description (13) which had a ROC AUC of 0.913 compared with 0.71-0.836 in other cohorts (24–26). In order to predict clinical outcomes after surgery for PA, Burrello et al. (14) created in 2019 the primary aldosteronism surgical outcome score. Designed to predict clinical outcomes, the PASO score is an option to the ARS. In our study, the PASO score was not found to be associated with complete clinical success. The ability to predict clinical cure is useful at the individual patient level to facilitate shared decision making and the informed consent process, but the relevance of these scoring systems for selecting patients for surgery should not be overestimated. For example, in the ARS validation group (12), even if a patient did not have any of these features, he still had a 25% chance of being completely cured by adrenalectomy.

Another interesting finding of our study is that we could identify one postoperative factor to be predictive of complete clinical success which is the pathologic nature of the resected adrenal gland. Patients with adrenal adenomas were the likeliest to be cured in our study. Wang et al. (18) found the same result as we did, Celen et al. (16) found that persistent hypertension after adrenalectomy was only favored by micronodular hyperplasia, while Proye et al. (26) did not found a statistically significant association between pathologic findings and hypertension cure. These results should prompt us to warn patients, in whom the diagnoses of hyperplasia has been confirmed by examination of the operative specimen, of the possibility of persistent hypertension and that complete resolution of hypertension is more unlikely.

Our study has few limitations. First, it is a retrospective review. Since the incidence of unilateral primary aldosteronism is rare, the time factor would make a prospective study difficult. We excluded analysis of biochemical outcomes because of missing data.

The fact that the majority of patients were not eligible for AVS, which may have introduced a selection bias and affected results, is a significant limitation of our study. Indeed, AVS is a technique which is not available in our country. It is undeniable that current recommendations agree that imaging alone is not sufficiently reliable to predict laterality, and that AVS is therefore the “gold standard”. In a review of the literature including 38 studies with 950 patients with PA, it was shown that if the diagnosis of laterality had been made solely on the basis of CT data, 14.6% of patients would have had an unnecessary adrenalectomy, 19.1% of patients would have been inappropriately excluded and finally 3.9% would have had an adrenalectomy on the wrong side (27). However, given the very limited feasibility of accurate and safe AVS and knowing the fact that it’s an invasive, relatively expensive procedure, requiring a specialized technical platform, some centers recommend nowadays alternative ways to subtype diagnoses of patients with PA. Recent studies showed that treatment of primary aldosteronism based on CT alone or AVS did not show significant differences in intensity of antihypertensive medication or clinical benefits for patients after 1 year of follow-up (28). However, despite the fact that the majority of our patients were selected on the basis of CT findings alone, the favorable clinical response rate was comparable to multiple studies carried out in expert centers that had used AVS to decide which side to operate on. Finally, current endocrine society guidelines suggest that patients aged < 35 years with marked PA and unilateral adrenal lesion on CT may not require AVS before proceeding to surgery which was the case in our study for 20% of the patients (8).

Strength of the present study is the relatively long follow-up time with a mean of almost two years (21 months). In fact, over longer follow-up, this may not be a permanent result in all patients. A recent meta-analysis showed that with each year of follow-up, the proportion of patients who maintain normal blood pressure without treatment decreases by 6.7% (9). This emphasizes the importance of prolonging the duration of postoperative follow-up in order to properly assess outcomes of the surgery.

## Conclusion

In our series, 64 patients (91%) had a favorable clinical response (complete and partial clinical success) to adrenalectomy. Cure of hypertension occurred in 48% of patients, whereas a more manageable hypertension occurred in 43% of patients. Absent clinical success was observed in only 9% of patients. We have established that the main predictors of complete resolution of hypertension in patients with UPA are absence of diabetes, low BMI, high plasma ARR and high ARS.

However, it is undeniable that the benefits of adrenalectomy do not apply to all patients with UPA without being able to accurately explain the underlying pathophysiological mechanisms of persistent hypertension. These findings highlight the increasing interest in determining preoperative predictors that could be used to assess patient eligibility for surgery. By obtaining data from larger numbers of patients and conducting the study prospectively in the future, we will be able to identify the precise mechanism of persistent hypertension and better understand its predictors.

In conclusion, most patients will benefit from laparoscopic adrenalectomy for UPA. However, identifying these subjects preoperatively is crucial, as the minority of them who will not benefit from adrenalectomy need to be fully warned about the risk of persistent or recurrent hypertension and that consequently they would need closer and longer follow-up after surgery.

## Data availability statement

The original contributions presented in the study are included in the article/supplementary material. Further inquiries can be directed to the corresponding author.

## Ethics statement

Ethical review and approval was not required for the study on human participants in accordance with the local legislation and institutional requirements. The patients/participants provided their written informed consent to participate in this study.

## Author contributions

Conception and Design: AS, MB and IN. Administrative support: HA, AD, KK, MC and RB. Data acquisition: AS, MB, SZ, SM and HB. Data analysis: MB and SZ. Manuscript writing: MB and AS. Final approval of manuscript: All authors. Critical revision of the manuscript: AS and MB.
